# γδ T Cells Modulate Myeloid Cell Recruitment but Not Pain During Peripheral Inflammation

**DOI:** 10.3389/fimmu.2019.00473

**Published:** 2019-03-18

**Authors:** Jelena Petrović, Jaqueline Raymondi Silva, Courtney A. Bannerman, Julia P. Segal, Abigail S. Marshall, Cortney M. Haird, Ian Gilron, Nader Ghasemlou

**Affiliations:** ^1^Department of Biomedical & Molecular Sciences, Queen's University, Kingston, ON, Canada; ^2^Department of Anesthesiology & Perioperative Medicine, Queen's University, Kingston, ON, Canada; ^3^Centre for Neuroscience Studies, Queen's University, Kingston, ON, Canada

**Keywords:** inflammation, neuroinflammation, behavior, formalin, complete Freund's adjuvant, post-surgical wound

## Abstract

Circulating immune cells, which are recruited to the site of injury/disease, secrete various inflammatory mediators that are critical to nociception and pain. The role of tissue-resident immune cells, however, remains poorly characterized. One of the first cells to be activated in peripheral tissues following injury are γδT cells, which serve important roles in infection, disease, and wound healing. Using a mouse line lacking these cells, we sought to identify their contribution to inflammatory pain. Three distinct models of peripheral inflammatory pain were used: intraplantar injection of formalin (spontaneous inflammatory pain), incisional wound (acute inflammatory pain), and intraplantar injection of complete Freund's adjuvant (chronic inflammatory pain). Our results show that absence of γδT cells does not alter baseline sensitivity, nor does it result in changes to mechanical or thermal hypersensitivity after tissue injury. Myeloid cell recruitment did show differential changes between models of acute and chronic inflammatory pain. These results were consistent in both male and female mice, suggesting that there are no sex differences in these outcomes. This comprehensive characterization suggests that γδT cells do not contribute to basal sensitivity or the development and maintenance of inflammatory pain.

## Introduction

The immune and nervous systems are intimately connected, particularly during inflammatory pain. Immune cells and their secreted mediators act on nociceptors in the periphery while neurons can modulate the inflammatory response (1–3). Peripheral inflammation is brought on by the well-orchestrated recruitment and activation of circulatory and tissue-resident immune cells, including mast cells, neutrophils, and macrophages (4, 5). These cells and their secreted mediators can alter nociceptor function/activity to induce nociceptor activation and/or peripheral sensitization, triggering increased responsiveness to noxious stimuli and pain hypersensitivity (1–7). While interactions between immune cells and nociceptors are essential in the pathophysiology of inflammatory pain, the cells/mediators controlling these outcomes remain poorly understood.

Various components of the immune system can bring about peripheral sensitization and pain hypersensitivity (1, 2, 5, 8–10). While the majority of this work has focused on secreted mediators, recent studies have identified specific contributions of immune cell subsets in mediating this pain sensitivity (11–18). Circulatory cells, including neutrophils and macrophages, have been shown to modulate inflammatory pain hypersensitivity (11, 13–16, 19). However, the role of tissue-resident cells is less understood. Recent work suggests skin-resident mast cells have no effect on inflammatory pain hypersensitivity (12), surprising given that these cells are known producers of inflammatory mediators that alter hypersensitivity (20–22). This brings about the question whether other tissue-resident immune cells play a role in inflammatory pain.

Previous work from our group evaluated the depletion of αβT cells (using TCRβ^−/−^ mice) and observed no role for these cells in inflammatory pain (11). γδT cells are less abundant than αβT cells, are the primary T cell population found in the gut mucosa and skin (23), and are absent in TCRδ^−/−^ mice (23). These skin-resident cells lie at the intersection of the innate and adaptive immune response and are among the first cells activated following tissue injury or viral/bacterial infection (24–31). Several studies show γδT cells contribute to the development of an inflammatory response in the gut, lungs, and spinal cord (among others), resulting in the recruitment and modulation of immune cells at the site of inflammation (32–37). We sought to identify what role γδT cells play in baseline sensitivity and inflammatory pain. Intraplantar injection of formalin or complete Freund's adjuvant, and plantar incisional wound were used to mimic human clinical inflammation (38, 39).

## Materials and Methods

This study was carried out in accordance with the recommendations of the ARRIVE (40) and Canadian Council on Animal Care guidelines. The protocol was approved by the Queen's University Animal Care Committee.

### Animals

B6.129P2-Tcrd^tm1Mom^/J mice (TCRδ^−/−^; Jackson Laboratory, Bar Harbor, ME) were backcrossed to C57BL/6J mice (Jackson Laboratory), generating wildtype, heterozygous and knockout littermates. All experiments were carried out using mice between 6 and 12 weeks of age, kept in a temperature- and humidity-controlled room with food and water provided *ad libitum*.

### Inflammatory Pain Models

Male and female TCRδ littermates were used for all experiments. Mice either received intraplantar injections with 20 μl of a 5% formalin solution, as described (41), intraplantar injections with 20 μl of complete Freund's adjuvant or a plantar incision, as described (11).

### Behavioral Assays

Licking/biting of the formalin-injected hindpaw was assessed in 5 min intervals over 60 min. Mechanical and thermal sensitivity, measured using von Frey, acetone, and Hargreaves tests, were carried out as described (11). Thermal sensitivity was assessed at specific temperatures using an air-cooled thermoelectric plate (TECA Corporation, Chicago, IL), by recording time to first response. A maximum cut-off of 30 s was used to prevent tissue damage. Only one measurement was taken at each temperature per experimental day to prevent learning behaviors; mice exhibiting learned-behaviors (e.g., scaling the enclosure) were excluded from further analysis (12).

### Immunofluorescence

Mice were anesthetized and sacrificed by transcardial perfusion with 2% paraformaldehyde in 0.1 M phosphate buffer. Ears were removed, post-fixed for 1 h, and cryoprotected in 30% sucrose. Serial 15 μm cryostat sections were incubated with anti-mouse TCRδ (1:100; Invitrogen, Waltham, MA) and anti-hamster IgG-FITC (1:200; BioLegend, San Diego, CA), and coverslipped for visualization using an AxioSkop2 fluorescent microscope (Carl Zeiss, Jena, Germany).

### Flow Cytometry

Immune cell infiltration/recruitment was assessed, as described (11). Footpads from male and female mice collected 24 h following incisional wound and CFA injection (*n* = 4/genotype/group) were stained using the following antibodies (BioLegend; 1:200): FITC anti–CD11b, PE-Cy7 anti–Ly6G, and APC/Fire750 anti–CD45. Flow cytometry was conducted on a CytoFLEX cytometer (Beckman Coulter, Indianapolis, USA) and analyzed using CytExpert software (Beckman Coulter).

### Statistical Analysis

All statistical analyses were carried out using SigmaPlot (Systat Software, San Jose, CA). Data are expressed as mean±SEM. One-way analysis of variance (ANOVA) was used for direct comparison between two or more groups, and two-way repeated-measures (RM) ANOVA used to assess change between groups over time, with *post-hoc* Tukey tests (*P* < 0.05).

## Results

γδT cells were visualized using immunohistochemistry and found to be present in the skin epidermal layer (Supplemental Figure 1) of TCRδ^+/−^ and TCRδ^+/+^ littermates but not in TCRδ^−/−^ mice, as we have previously shown (42). We first assessed whether loss of γδT cells causes a change to baseline mechanical or thermal sensitivity in male (*n* = 14–22) or female (*n* = 10–18) mice (Supplemental Figure 1). Mechanical sensitivity, measured as the 50% threshold (*P* ≥ 0.276, one-way ANOVA), and thermal sensitivity, assessed using the acetone (*P* ≥ 0.669, one-way ANOVA) and Hargreaves radiant heat tests (*P* ≥ 0.086, one-way ANOVA), respectively, did not show any difference between TCRδ littermates in males or females. The hot/cold plate test was used to identify differences in noxious thermal response using fixed temperatures between 0 and 55°C. Male (*n* = 7–15) and female (*n* = 6–17) littermates did not show any significant differences in time to first response, while there was a group effect in females between all three strains at 55°C (*P* = 0.039), no significant differences were observed between genotypes (*post-hoc* Tukey test *P* = 0.063).

**Figure 1:**
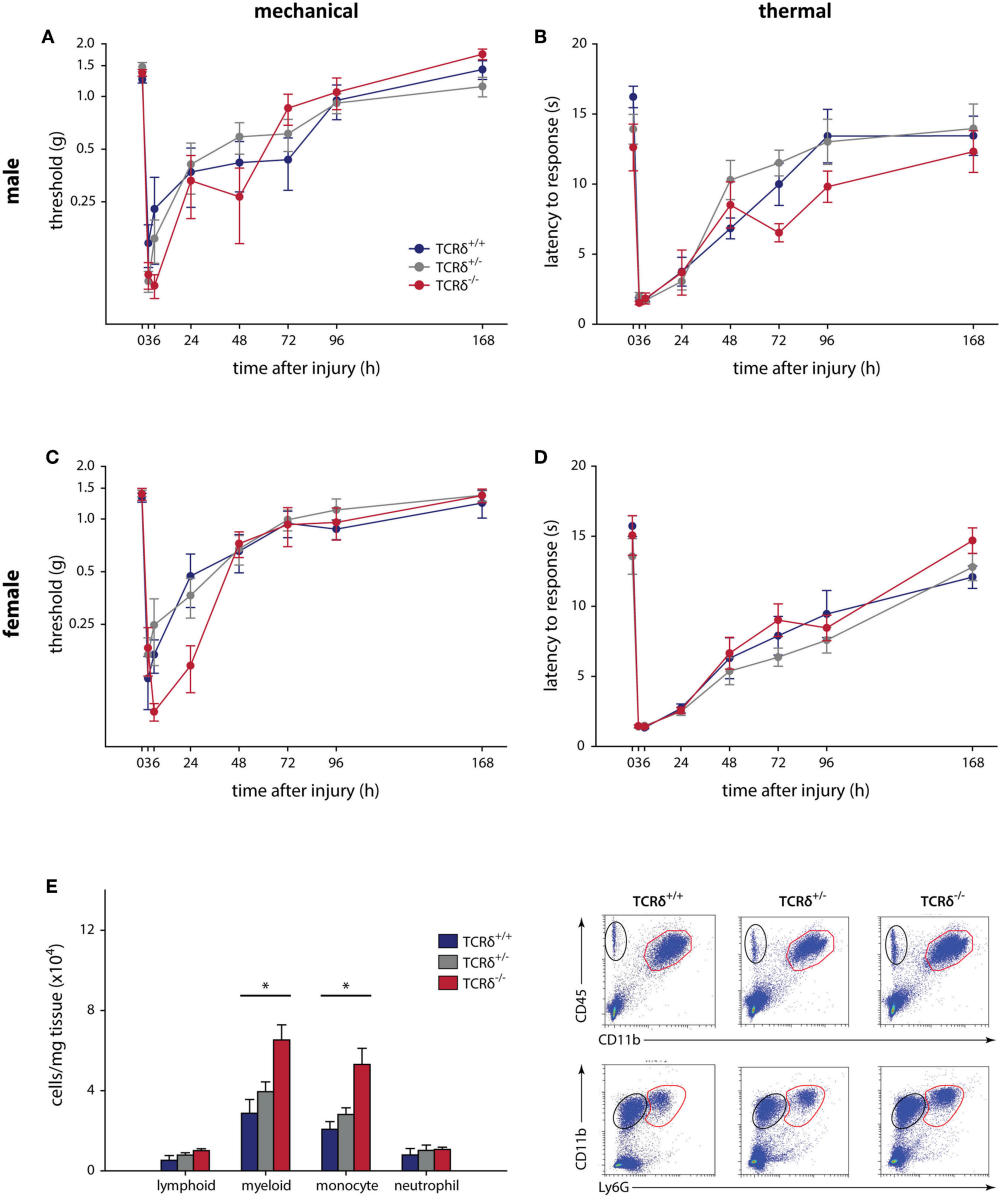
γδT cells do not contribute to mechanical and thermal hypersensitivity after incisional wound, and do not affect immune cell recruitment. Male TCRδ littermates (*n* = 7–10 per genotype) did not exhibit differences in mechanical thresholds (**A**; *P* = 0.064, two-way RM-ANOVA), measured with von Frey monofilaments, or heat hypersensitivity (**B**; *P* = 0.215, two-way RM-ANOVA), measured as the latency of response to a radiant heat stimulus. A similar effect was observed in female TCRδ littermates (*n* = 8–12 per genotype) for both mechanical (**C**; *P* = 0.942, two-way RM-ANOVA) and thermal (**D**; *P* = 0.675, two-way RM-ANOVA) hypersensitivity. **(E)** Loss of γδT cells in TCRδ^−/−^ mice significantly reduces myeloid immune cell (CD45^+^CD11b^+^; top row, red) and monocyte (CD45^+^CD11b^+^Ly6G^−^; bottom row, red) recruitment/infiltration into the inflamed hindpaw 24 h after incisional wound, but does not affect lymphoid cells (CD45^+^CD11b^−^; top row, black) or neutrophils (CD45^+^CD11b^+^Ly6G^+^; bottom row, black) relative to TCRδ^−/+^ and TCRδ^+/+^ littermates (^*^P < 0.05, one-way ANOVA). Representative flow cytometry plots are shown (*n* = 4/genotype).

The contribution of γδT cells to the inflammatory pain response was assessed using standard assays, with 2–4 cohorts of littermates used in all tests. The contribution of γδT cells to acute inflammatory pain outcomes using the formalin test (Supplemental Figure 2), a model of non-reflexive pain lasting ~1 h (41, 43), showed no effect in male (*n* = 6–9) and female (*n* = 8–13) TCRδ mice when measured in 5 min intervals (*P* ≥ 0.353, two-way RM-ANOVA), nor in total response time during the acute and tonic stages (*P* ≥ 0.338, one-way ANOVA). We next considered the contribution of γδT cells to the development and maintenance of acute inflammatory pain following plantar incisional wound, a model of post-surgical pain that often resolves within 3–4 days. Male (*n* = 7–10) and female (*n* = 8–12) littermates showed no difference between mechanical (*P* ≥ 0.064, two-way RM-ANOVA) and heat (*P* ≥ 0.215, two-way RM-ANOVA) hypersensitivity, assessed over 7 days (Figure 1). While there were no behavioral differences, significantly increased myeloid cells were observed in the hindpaws of TCRδ^−/−^ mice relative to TCRδ^+/+^ and TCRδ^+/−^ littermates after incisional wound (*n* = 4/group, one-way ANOVA, *P* < 0.05; Figure 1E; Supplemental Figure 3A).

**Figure 2:**
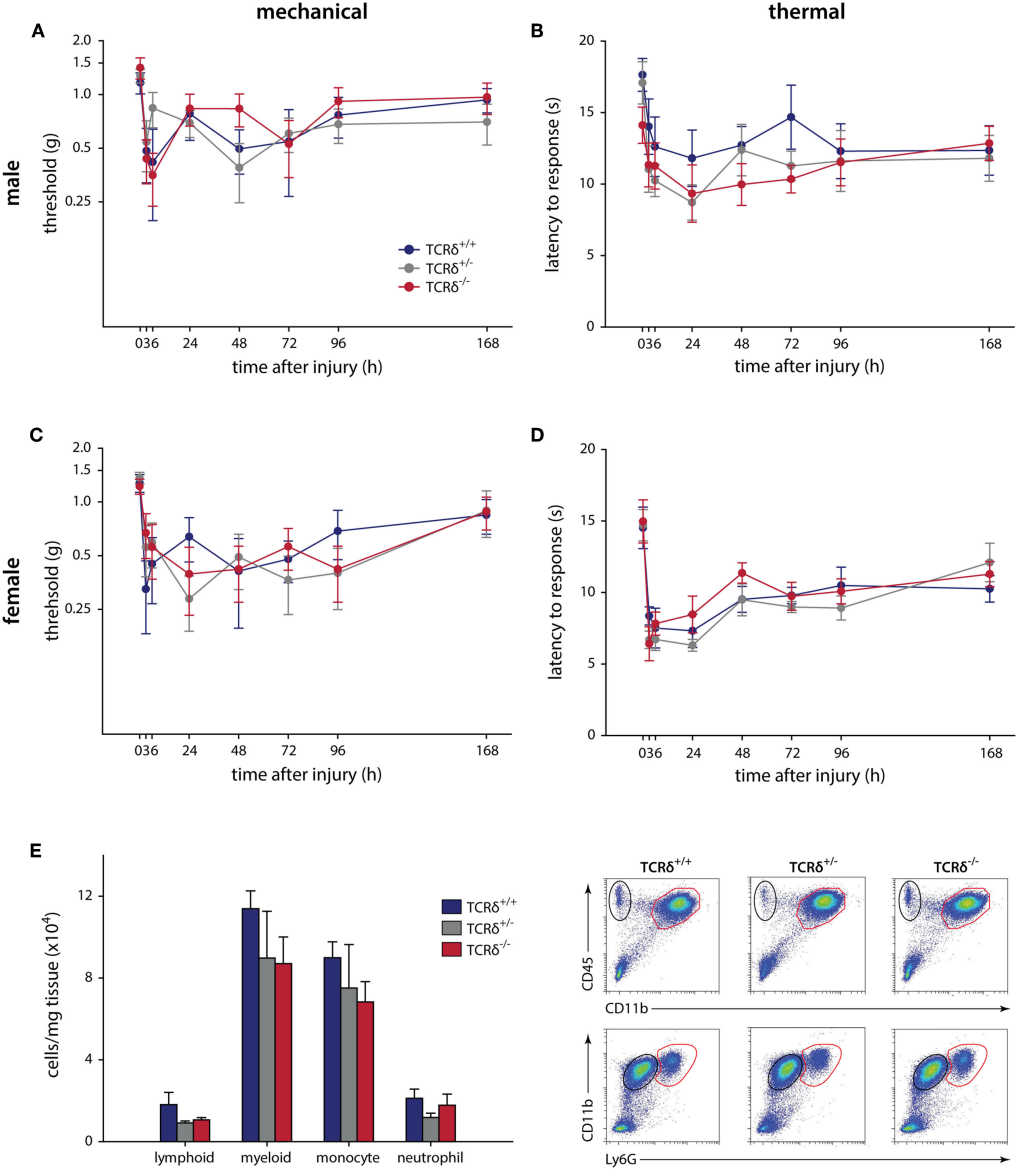
CFA-induced hypersensitivity is unaffected by loss of γδT cells, while myeloid cell recruitment is significantly affected. TCRδ littermates received intraplantar injections of complete Freund's adjuvant and pain outcomes were measured over 7 days. Differences in mechanical (**A**; *P* = 0.226, two-way RM-ANOVA) or thermal (**B**; *P* = 0.943, two-way RM-ANOVA) in male mice (*n* = 9–12 per genotype). Female littermates (*n* = 6–9 per genotype) also did not show any differences in mechanical (**C**; *P* = 0.530, two-way RM-ANOVA) or thermal (**D**; *P* = 0.857, two-way RM-ANOVA) responses. **(E)** Loss of γδT cells significantly reduced immune cell recruitment/infiltration into the inflamed hindpaw 24 h after intraplantar injection of complete Freund's adjuvant. While the percentage of lymphoid cells (CD45^+^CD11b^−^) and neutrophils (CD45^+^CD11b^+^Ly6G^+^) is unaffected, there is a significant decrease in the percentage of myeloid cells (CD45^+^CD11b^+^) and monocytes (CD45^+^CD11b^+^Ly6G^−^) in the footpad of TCRδ^−/−^ mice relative to TCRδ^−/−^ and TCRδ^−/−^ littermates. Representative flow cytometry plots are shown (*n* = 4/genotype).

We finally carried out intraplantar injection of CFA to determine whether γδT cells contribute to chronic inflammatory pain, an example of granulomatous inflammation that does not resolve (44). Similar to the formalin and incisional wound models, no significant effects were observed in male (*n* = 9–12) or female (*n* = 6–9) littermates when assessed for mechanical (*P* ≥ 0.226, two-way RM-ANOVA) and thermal (*P* ≥ 0.857, two-way RM-ANOVA) hypersensitivity over 7 days (Figure 2). Behavioral analysis was carried out until day 7 to minimize any potential systemic effect caused by intraplantar CFA injection (45). The inflammatory response in TCRδ^−/−^ mice showed a trend toward decreased myeloid cells compared littermate controls, though this was not significant (*n* = 4/group, *P* ≥ 0.336, one-way ANOVA; Figure 2E; Supplemental Figure 3B).

## Discussion

Understanding how immune cells contribute to the development and maintenance of pain will be crucial to the development of safe and efficacious therapeutics for the treatment of inflammatory pain. Our group previously identified the contribution of lymphocytes to inflammatory pain, using cell-specific strategies to deplete neutrophils, non-neutrophil myeloid, and αβ T cells (11). While only non-neutrophil myeloid cells were found to alter behavioral outcomes, and only after incisional wound, the role of most tissue-resident cells to inflammatory pain remains unknown. We therefore set out to determine the contribution of γδT cells to the development and maintenance of acute and chronic inflammatory pain.

Our work started by showing that loss of γδT cells did not have an effect on basal sensitivity, an important finding given that these cells are known to interact with sensory fibers during inflammation (46). Using three models of peripheral inflammatory pain, including intraplantar injection of formalin/CFA and incisional wound, we found that γδT cells do not alter mechanical or thermal sensitivity during inflammation. The pain outcomes observed in TCRδ wildtype and heterozygous animals in the formalin, incision, and CFA models matches that observed in C57BL/6 mice in previous studies by our group and others (11, 47, 48). Loss of γδT cells did, however, reduce the recruitment of myeloid cells to the hindpaw after incision; recruitment of myeloid cells after CFA injection was paradoxically increased, though this was not significant. While our results suggest that γδT cells do not contribute to inflammatory pain hypersensitivity, only three models of disease were used. Assessing the role of these cells may show an important effect in pain outcomes following bacterial infection, where they are known to have an effect in both skin (49, 50) and lung (46, 51, 52), or in models of inflammatory bowel disease (53–55). We hypothesize this to be possible due to the high number of these cells in the lungs and lining the gut mucosa. Although our results do not show an effect for γδT cells in the nociceptive response following peripheral inflammation, these cells may still play an important role in itch and other skin pathologies and could prove useful in identifying novel underlying cellular and molecular mechanisms.

While there are conflicting results in the literature as to the function of γδT cells in the modulation of inflammation, we now show a divergent role for these cells in the recruitment of myeloid cells in the footpad following injury/inflammation. Our results suggest the immunomodulatory role of γδT cells depends on the type of immune response necessary: a strong inflammatory response is present in the CFA-injected footpad, while a less pronounced inflammation is found following incisional wound. Recent evidence now points to an important role for γδT cells in the recruitment of myeloid cells, including neutrophils, monocytes, and macrophages (26, 34, 56–58), though these responses are dependent on the site and type of injury/disease. This could explain the lack of effect in TCRδ^−/−^ mice treated with CFA. Other studies, however, have found that these cells are either not required or negatively regulate skin inflammation (26, 59, 60), as we have observed following incisional wound. This may be due to the fact that incisional wound, like burn injuries, resolve themselves. The increased myeloid cell infiltration after burn-induced wound in TCRδ^−/−^ mice helps to initiate the proliferative phase of wound healing (26); it is therefore possible that γδT cells reduce myeloid cell recruitment in models of resolving inflammation but increase recruitment in chronic inflammatory states. γδT cells produce various inflammatory mediators (e.g., interferon (IFN)-γ, IL-17, TNF-α, granzymes, and insulin-like growth factor-1) after injury (61), keratinocyte and fibroblast growth factors (KGFs and FGFs) are two major classes of mediators secreted by these cells. While several FGF family members have been found to directly activate sensory neurons (3, 62, 63), KGFs are not known to affect sensory neuron activity, though keratinocytes themselves have recently been implicated in modulating nociception (64, 65), itch (66, 67), and mechanosensitivity (68, 69). While our results demonstrate that γδT cells do not contribute to inflammatory nociception, this is limited by our use of these three models. This first study of the function of γδT cells in mediating pain outcomes is limited to inflammation in the hindpaw; we speculate that future work examining the function of these cells in other models of pain/nociception may yet identify a role for these cells.

## Data Availability

The datasets generated for this study are available on request to the corresponding author.

## Author Contributions

JP, JRS, IG, and NG contributed to the conception and design of the study. JP performed all behavioral analysis. JRS, CAB, JPS, ASM, and CMH performed histology and flow cytometry experiments. JP and NG performed the statistical analysis. JP wrote the first draft of the manuscript. JRS, IG, and NG wrote sections of the manuscript. All authors contributed to manuscript revisions, read, and approved the submitted version.

### Conflict of Interest Statement

The authors declare that the research was conducted in the absence of any commercial or financial relationships that could be construed as a potential conflict of interest.

## Supplementary Material

Supplemental Figure 1 Absence of γδT cells does not affect basal mechanical or thermal sensitivity. **(A)** Sections of the ear from wildtype, heterozygous, and knockout TCRδ mice (*n* = 3–4/group) immunostained for γδT cells using an antibody recognizing the δ T cell receptor subunit. Representative micrographs show γδT cells are present in TCRδ^+/+^ and TCRδ^+/−^ mice; these cells were never visualized in TCRδ^−/−^ littermates. **(B)** Mechanical thresholds, measured as the von Frey monofilament corresponding to a 50% response, is not affected by loss of γδT cells in male (*P* = 0.402, one-way ANOVA; *n* = 17–22 per genotype) or female (*P* = 0.276, one-way ANOVA; *n* = 15–18 per genotype) mice. **(C)** Cold thermal responses were assessed using the acetone test, measured as total response time (e.g., licking and biting of the affected hindpaw), was not different between male (*P* = 0.669, one-way ANOVA; *n* = 14–16 per genotype) or female (*P* = 0.758, one-way ANOVA; *n* = 10–15 per genotype) littermates. **(D)** Thermal heat hypersensitivity was measured as the latency to response following stimulation of the hindpaw by a radiant heat source. No differences were observed in either male (*P* = 0.086, one-way ANOVA; *n* = 17–19 per genotype) or female (*P* = 0.679, one-way ANOVA; *n* = 15–18 per genotype) mice. **(E)** No differences were observed in latency to paw withdrawal (e.g., flinch) using the hot and cold plate test in male TCRδ littermates (*P* ≥ 0.193, one-way ANOVA; *n* = 6–15 per genotype) at any of the temperatures examined. **(F)** Female mice assessed for latency to first response did not exhibit differences at 0, 50, or 52°C (*P* ≥ 0.099, one-way ANOVA; *n* = 6–17 per genotype). While there was a significant group effect for genotype at 55°C (*P* = 0.039, one-way ANOVA), *post-hoc* Tukey analysis was not significant between the three groups (*P* ≥ 0.063). Graphs show mean ± SEM, scale bar = 50 μm.

Supplemental Figure 2 Response to formalin is unaffected by absence of γδT cells. TCRδ littermates were injected with formalin and the response time measured over 60 min. Male mice (*n* = 6–9 per genotype) did not show an effect over the duration of response (**A**; *P* = 0.403, two-way RM-ANOVA) or during acute and tonic phases (**B**; *P* ≥ 0.400, one-way ANOVA). Female mice (*n* = 8–13 per genotype) also did not show a significant effect over the duration of response (**C**; *P* = 0.353, two-way RM-ANOVA) or in acute/tonic phases (**D**; *P* ≥ 0.338, one-way ANOVA).

Supplemental Figure 3 Percentage of immune cells in the hindpaw of mice 24 h after inflammatory injury, assessed by flow cytometry. **(A)** Loss of γδT cells results in a significantly increased percentage of myeloid cells and monocytes, relative to TCRδ^+/+^ and TCRδ^+/−^ mice. **(B)** There are no significant differences in the percentage of immune cells in the hindpaws of TCRδ^+/+^, TCRδ^+/−^, and TCRδ^−/−^ mice.
