# Supplementary figures and images for: γδ T Cells Modulate Myeloid Cell Recruitment but Not Pain During Peripheral Inflammation
Source: Front Immunol. 2019 Mar 18;10:473. doi: 10.3389/fimmu.2019.00473 (PMC6431614; doi:10.3389/fimmu.2019.00473)

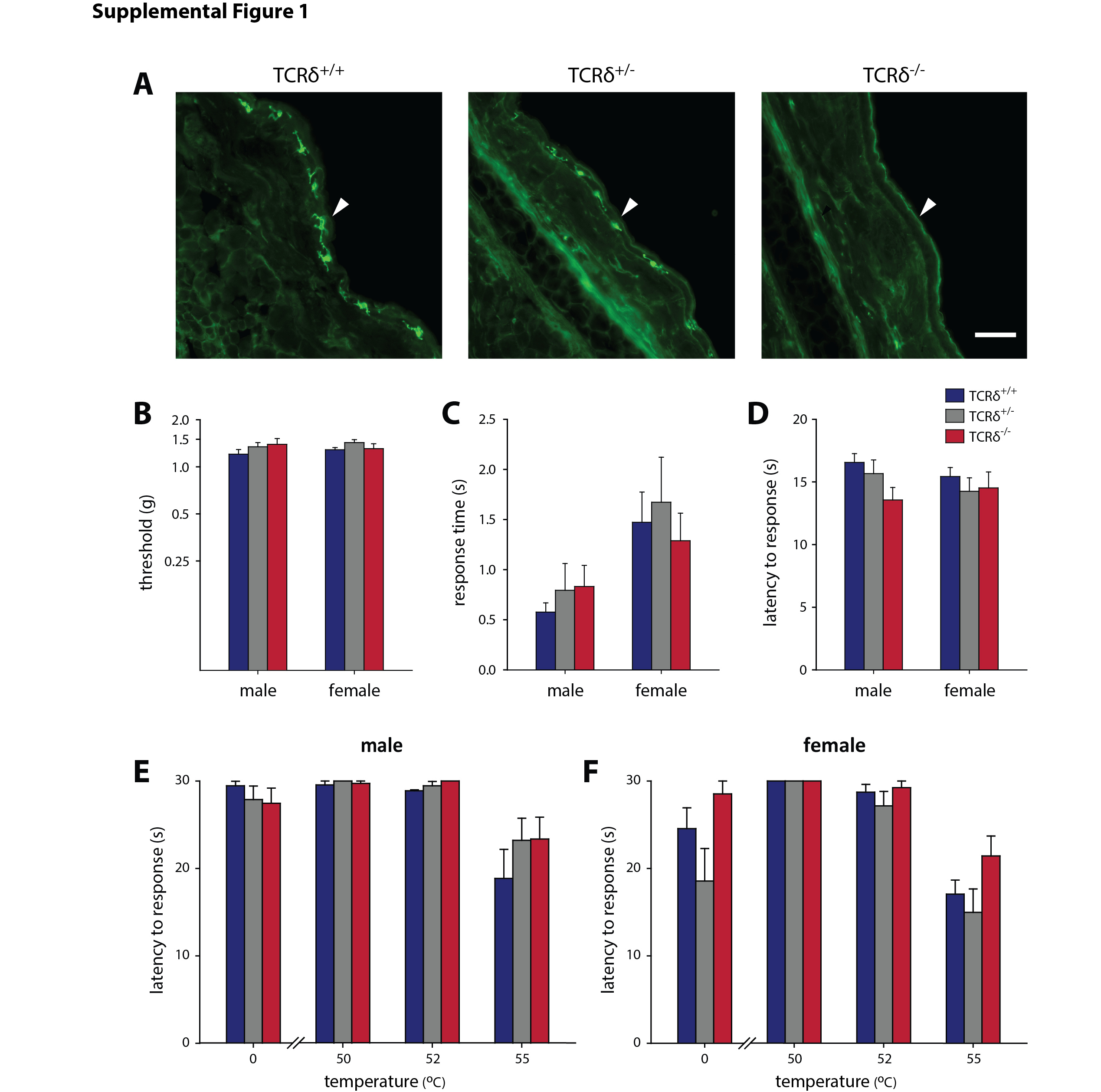

Supplement: Supplemental Figure 1 — Absence of γδT cells does not affect basal mechanical or thermal sensitivity. (A) Sections of the ear from wildtype, heterozygous, and knockout TCRδ mice (n = 3–4/group) immunostained for γδT cells using an antibody recognizing the δ T cell receptor subunit. Representative micrographs show γδT cells are present in TCRδ+/+ and TCRδ+/− mice; these cells were never visualized in TCRδ−/− littermates. (B) Mechanical thresholds, measured as the von Frey monofilament corresponding to a 50% response, is not affected by loss of γδT cells in male (P = 0.402, one-way ANOVA; n = 17–22 per genotype) or female (P = 0.276, one-way ANOVA; n = 15–18 per genotype) mice. (C) Cold thermal responses were assessed using the acetone test, measured as total response time (e.g., licking and biting of the affected hindpaw), was not different between male (P = 0.669, one-way ANOVA; n = 14–16 per genotype) or female (P = 0.758, one-way ANOVA; n = 10–15 per genotype) littermates. (D) Thermal heat hypersensitivity was measured as the latency to response following stimulation of the hindpaw by a radiant heat source. No differences were observed in either male (P = 0.086, one-way ANOVA; n = 17–19 per genotype) or female (P = 0.679, one-way ANOVA; n = 15–18 per genotype) mice. (E) No differences were observed in latency to paw withdrawal (e.g., flinch) using the hot and cold plate test in male TCRδ littermates (P ≥ 0.193, one-way ANOVA; n = 6–15 per genotype) at any of the temperatures examined. (F) Female mice assessed for latency to first response did not exhibit differences at 0, 50, or 52°C (P ≥ 0.099, one-way ANOVA; n = 6–17 per genotype). While there was a significant group effect for genotype at 55°C (P = 0.039, one-way ANOVA), post-hoc Tukey analysis was not significant between the three groups (P ≥ 0.063). Graphs show mean ± SEM, scale bar = 50 μm. [file Image_1.TIF]

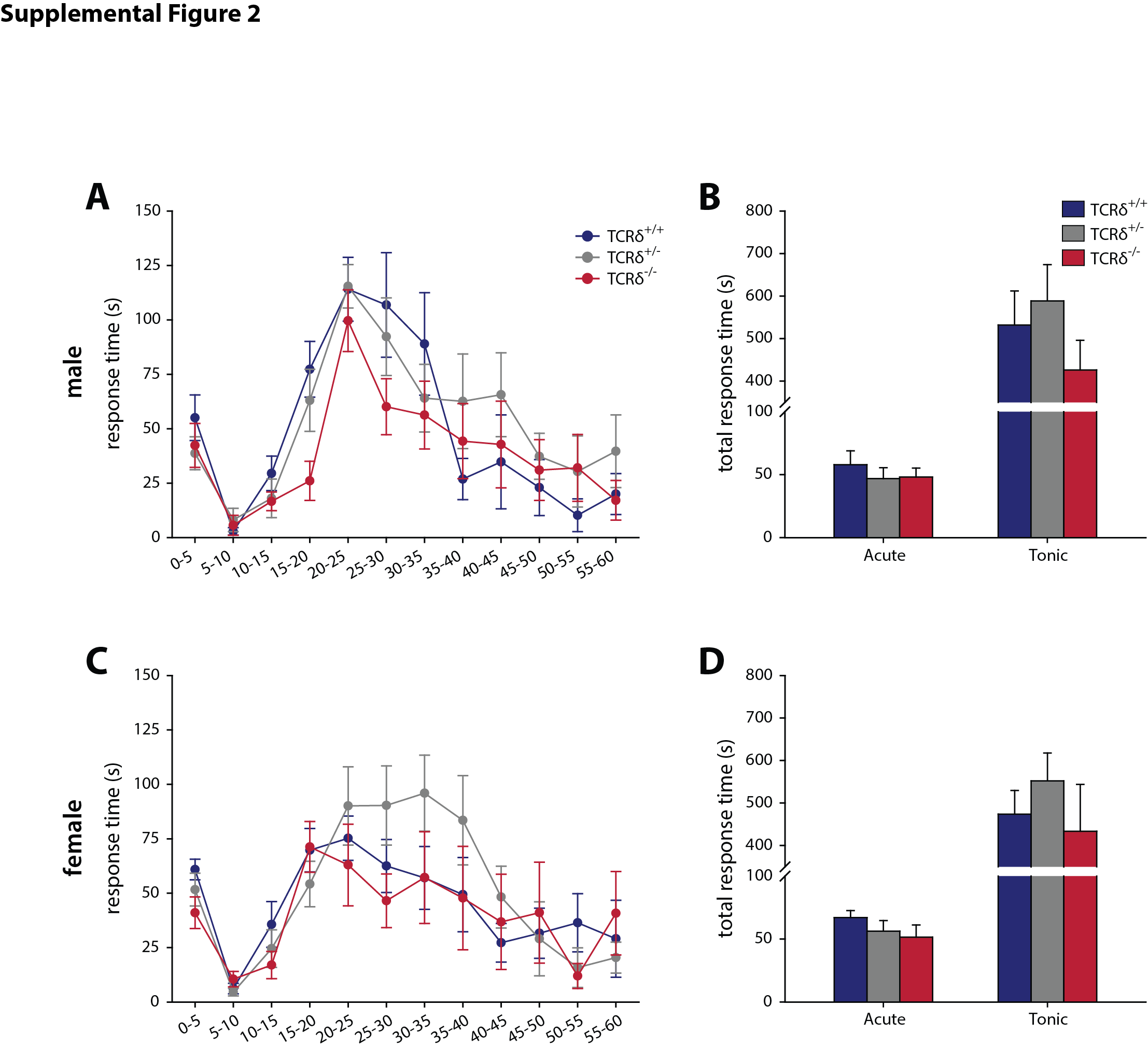

Supplement: Supplemental Figure 2 — Response to formalin is unaffected by absence of γδT cells. TCRδ littermates were injected with formalin and the response time measured over 60 min. Male mice (n = 6–9 per genotype) did not show an effect over the duration of response (A; P = 0.403, two-way RM-ANOVA) or during acute and tonic phases (B; P ≥ 0.400, one-way ANOVA). Female mice (n = 8–13 per genotype) also did not show a significant effect over the duration of response (C; P = 0.353, two-way RM-ANOVA) or in acute/tonic phases (D; P ≥ 0.338, one-way ANOVA). [file Image_2.TIF]

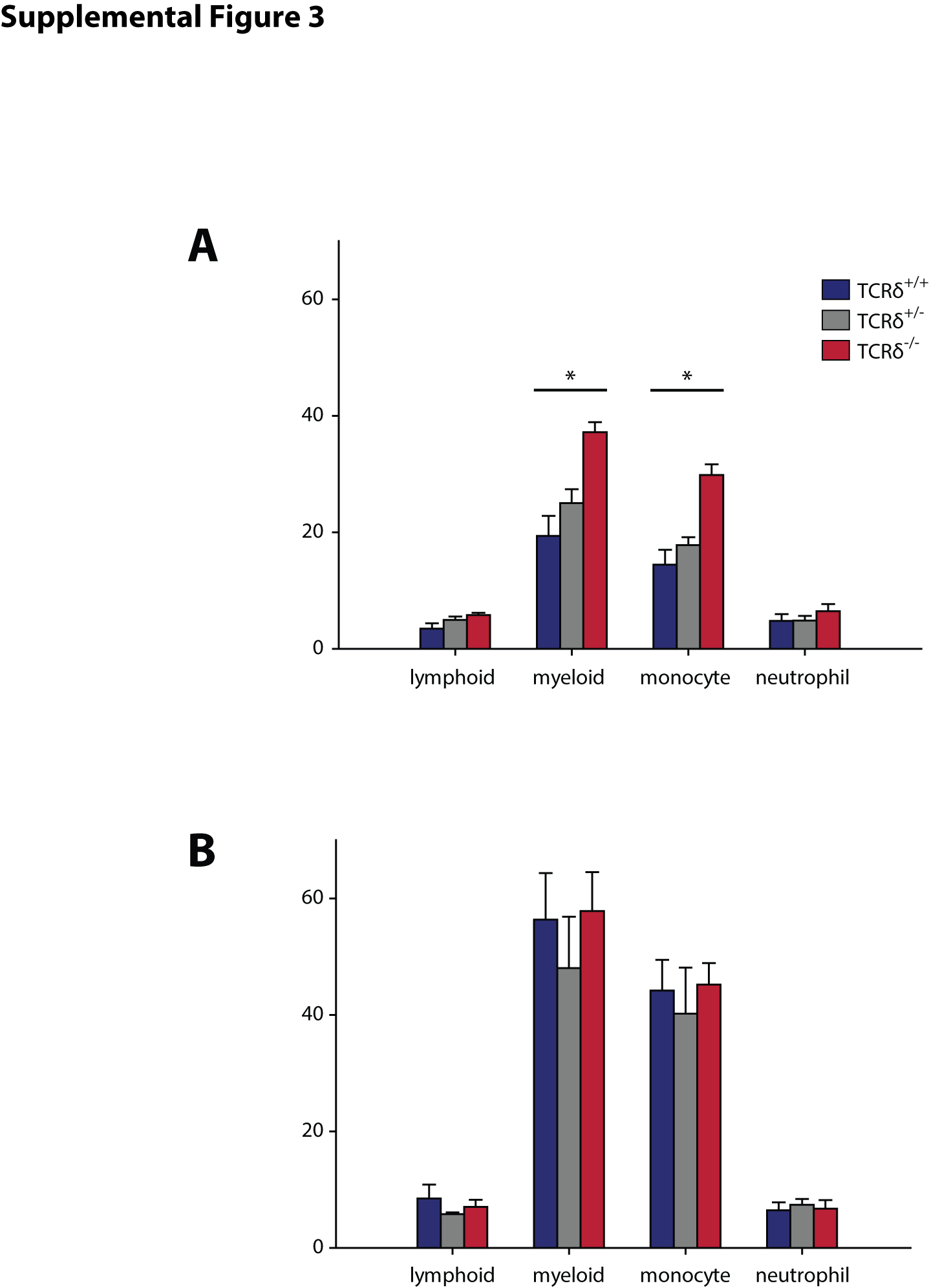

Supplement: Supplemental Figure 3 — Percentage of immune cells in the hindpaw of mice 24 h after inflammatory injury, assessed by flow cytometry. (A) Loss of γδT cells results in a significantly increased percentage of myeloid cells and monocytes, relative to TCRδ+/+ and TCRδ+/− mice. (B) There are no significant differences in the percentage of immune cells in the hindpaws of TCRδ+/+, TCRδ+/−, and TCRδ−/− mice. [file Image_3.TIF]
